# Quantitative Evaluations with 2d Electrical Resistance Tomography in the Low-Conductivity Solutions Using 3d-Printed Phantoms and Sucrose Crystal Agglomerate Assessments

**DOI:** 10.3390/s21020564

**Published:** 2021-01-14

**Authors:** Guruprasad Rao, Muhammad Awais Sattar, Radosław Wajman, Lidia Jackowska-Strumiłło

**Affiliations:** Institute of Applied Computer Sciences, Lodz University of Technology, 90-924 Lodz, Poland; muhammad.sattar@p.lodz.pl (M.A.S.); radoslaw.wajman@p.lodz.pl (R.W.); lidia.jackowska-strumillo@p.lodz.pl (L.J.-S.)

**Keywords:** 2D electrical resistance tomography, low-conductivity solutions, crystallization, inverse imaging

## Abstract

Crystallization is a significant procedure in the manufacturing of many pharmaceutical and solid food products. In-situ electrical resistance tomography (ERT) is a novel process analytical tool (PAT) to provide a cheap and quick way to test, visualize, and evaluate the progress of crystallization processes. In this work, the spatial accuracy of the nonconductive phantoms in low-conductivity solutions was evaluated. Gauss–Newton, linear back projection, and iterative total variation reconstruction algorithms were used to compare the phantom reconstructions for tap water, industrial-grade saturated sucrose solution, and demineralized water. A cylindrical phantom measuring 10 mm in diameter and a cross-section area of 1.5% of the total beaker area was detected at the center of the beaker. Two phantoms with a 10-mm diameter were visualized separately in noncentral locations. The quantitative evaluations were done for the phantoms with radii ranging from 10 mm to 50 mm in demineralized water. Multiple factors, such as ERT device and sensor development, Finite Element Model (FEM) mesh density and simulations, image reconstruction algorithms, number of iterations, segmentation methods, and morphological image processing methods, were discussed and analyzed to achieve spatial accuracy. The development of ERT imaging modality for the purpose of monitoring crystallization in low-conductivity solutions was performed satisfactorily.

## 1. Introduction

Industrial process tomography and monitoring is an important field of applied research, which uses many types of modern sensors to monitor and evaluate the current state of the physical or chemical processes. It has applications in a wide range of industries such as food, pharmaceuticals, and the petroleum industry. Based on the underlying physical principle involved and characteristics (i.e., offline, online, or in situ) of the process, a variety of process analytical technology (PAT) sensors are utilized in process control systems. Crystallization process monitoring as well as crystal morphology assessments are performed using various PAT modalities such as light reflection [1] and confocal microscopy [2] for morphological measurements and CCD cameras [3], and hot stage microscopy [4] for observation of suspensions. The 3D surface measurements to measure particle size [5], Raman spectroscopy, ultraviolet (UV), mid-IR spectroscopy, and focused beam reflection measurements (FBRM) [6] to observe polymorphic transformations are some of the advanced PAT technologies in crystallization monitoring. Various imaging techniques involving the mapping of convection, temperature, and concentration for the measurement of growth rate and micromorphology of crystal surfaces have been utilized [7] for this purpose. Additionally, different 3D imaging techniques, such as optical tomography, magnetic resonance imaging (MRI), radiographic computed axial tomography (CAT), shadowgraphic tomography, and interferometric tomography, have been utilized for the crystal analyses [7] to observe the crystal growth from solutions. Analytical process technologies for crystallization process monitoring and control, such as attenuated total reflectance Fourier transform infrared (ATR-FTIR) and UV-visible spectroscopy, have successfully separated solid and liquid phases [8]. Many imaging techniques for the morphology evaluation of crystals use microscopic samples in the offline system for particle characterization [9]. However, online PAT imaging techniques, such as Electrical Capacitance Tomography (ECT) [10,11,12,13] and ultrasound tomography [14,15], have proved to be very useful tomographic techniques to visualize the fluids and dense phases or separate the solutions depending on concentrations.

Electrical resistance tomography (ERT) as an imaging modality has been used in various research domains. In the field of geological evaluations, it is used to detect the groundwater alluvium and analyze the rock structures [16], large historical buildings [17] and to observe the subsurface solute transport within the soil [18,19]. In biomedical applications, ERT is utilized to detect pulmonary regions using impedance plethysmography techniques [20]. In chemical engineering, ERT is used to assess different pipes and various storage tanks for observing the liquid–solid and gas–liquid–solid processes [21]. These applications involve different spatial sizes of the target region of interest and varied conductivity profiles. Even though it is challenging to do ERT imaging in low-conductivity environments [22], many applications have been demonstrated, such as imaging composite structures [23] and bone cement [24].

For pharmaceutical applications, developing new types of crystallizers and solvent/solute monitoring is an active research field [25]. As the pharmaceutical crystallization goes from batch to continuous [26], online process monitoring in the pharmaceutical industry will also be of high importance. The development of ERT for pharmaceutical products [27] and multiphase monitoring in pharmaceutical processes [28] has many distinct advantages. In addition to the specifically-designed sensors to observe the pharmaceutical products [27,28], the crystallization informatics system, which implements direct control over the nucleation in the supersaturated solutions [29], has also been developed. This indicates the utility of the ERT as a PAT technology [30] in various other industries, such as industrial sugar mills [31] and milk powder crystal manufacturing [32], for decision support and monitoring.

Monitoring and control of the crystallization processes are inherently challenging due to the different types of crystallization techniques involved, such as cooling crystallization, antisolvent crystallization, and reactive crystallization. Each of these types requires a different type of process analytical tool or method for the process progress evaluation. Advances have been made in the modeling, monitoring, and control of the crystals [33] and one-dimensional PAT evaluations, involving combined cooling and antisolvent crystallization by measuring temperatures and analyzing microscopic images of crystals [34], along with observation of kinetics [35]. Electrical resistance tomography is one of the novel process analytical tools used to monitor the crystallization process. The 2D ERT allows us to detect and visualize the conductivity distribution inside the chemical reactor using electrical voltage or current measurements acquired from the periphery of the reactor. This is utilized to evaluate the different stages of the process [36].

To produce value-added products in chemical industries, PAT is particularly utilized in large-scale reactors and tanks. The large-scale chemical process industries have used the ERT extensively to monitor the unbaffled stirred tanks and reactors to check the solid and liquid distribution [37,38,39]. A multilayered ERT system has been used to visualize the dense solid particles in a solid–liquid stirred tank [37] and observe precipitation reactions [40]. Large-scale crystallization monitoring and control to improve predictability and robustness of the chemical reaction products can also be performed using ERT [33].

**Figure 1:**
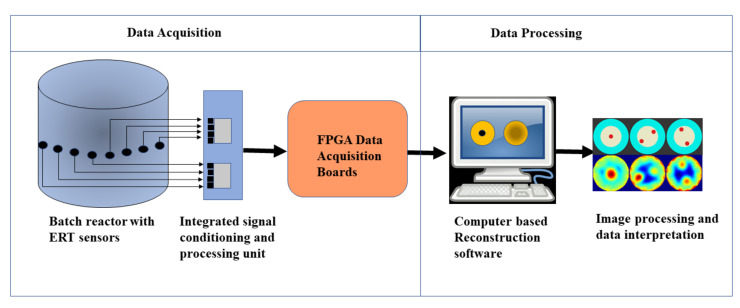
Schematic of the electrical resistance tomography (ERT) data acquisition and data processing system.

The ERT system primarily consists of two main sections, as shown in Figure 1. They are the data acquisition section and the data processing section. These sections contribute to the chain of evaluations on which the control of the chemical crystal reactions can be achieved. The quantitative spatial accuracy of the image object determined using the ERT imaging modality depends on various factors in this imaging setup.

The main challenge to utilize ERT in the antisolvent crystallization process is the low conductivity of the solutions involved [41]. It affects the ability to perform quantitative evaluations and to implement the control system in the nonionic solutions [22,36]. The objective of this work was to test the accuracy of the newly developed sensor of the ERT system for the accurate representation of nonconductive objects in the low-conductivity solutions. This was evaluated by placing small phantoms in the central and peripheral locations in the reactor. Tap water, industrial-grade saturated sucrose solution, and demineralized water were compared. Three ERT image reconstruction algorithms were evaluated for six different phantoms. Four segmentation methods were tested. The color channels from the image were extracted, and binarization on the green channel (G-Channel) was applied to investigate the region of interest within the reactor. The sugar crystal agglomerations in demineralized water using ERT were tested quantitatively. The experimental setup, progression, and test objectives are discussed in detail in Section 3.3. The novelty of the work lies in the identification and measurement validation of the factors affecting the accuracy in the spatial domain for ERT measurements in low-conductivity solutions.

## 2. ERT Imaging

### 2.1. Modeling and Simulation Studies in ERT

**Figure 2:**
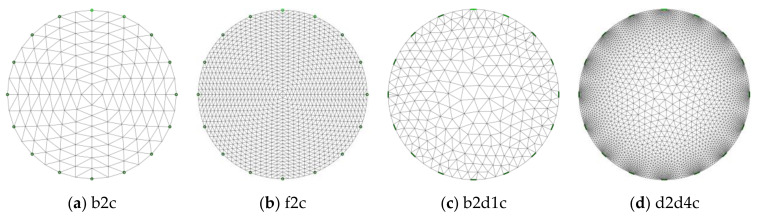
**Finite Element Model** (FEM) mesh generated using EIDORS at various mesh densities.

Prior to advancements in high-speed computation capabilities with modern computers, qualitative imaging in process engineering was performed using the ERT [42]. Many simulation studies in ERT data acquisition hardware and reactors have been performed to extract quantitative information. The study of the nature of current or voltage fields using electrode array simulation [43] and the complex conductivity distributions in the ERT images using the Finite Element Model (FEM) mesh [44] improved the analysis of the process data. Also, the entire batch reactor models were simulated to interpret the process progression [45]. Together with sensor simulations, the comparisons with experiments were performed [46] to observe the voltage changes. The studies involving quantitative inverse modeling for the cylindrical object using various FEM meshes [47] and improvement of sensitivity matrix for ERT [48] and ECT [49] showed interdependence of model design and accuracy of the estimations. In the study by [50], two separated phantoms of radius 2 cm were evaluated using iterative Gauss–Newton (GN) methods and segmented with the Otsu and adaptive threshold segmentation method at various iterations. Figure 2 shows the different FEM meshes generated using EIDORS [51,52,53]. The mesh elements can be of equal sizes, as shown in Figure 2a,b, or densely populated around the electrode, as shown in Figure 2c,d, which corresponds better to sensor spatial sensitivity. The nomenclature for the meshes is a standard used in EIDORS software.

### 2.2. Reconstruction Methods

To find conductivity distribution using reconstruction methods within a cylindrical reactor plane is essentially an ill-posed problem. It consists of two parts, a forward problem and an inverse problem. In the forward problem, the electrical field, the boundary conditions, and the assumed conductivity distribution is obtained for the circular geometrical region. In the inverse problem, the conductivity distributions are estimated. This is achieved by minimizing the differences between the calculated and the measured electrical signals on the electrodes [54]. The inverse problems do not have a unique solution; hence, a small change in the data can cause large changes in the resulting mathematical solution/reconstructed images [55]. In this work, linear back projection (LBP), Gauss–Newton (GN), and total variation (TV) methods were used. The noser prior defined in EIDORS software was used to generate initial conditions for the GN, and *TV prior* was used for the TV algorithm. The point electrode model was used. The fmdl.electrode(idx).z_contact denoting the contact impedance was set at 0.

In ERT, the straight line for back projection cannot be used, as any single change in the object affects all the current measurements. All these current values are utilized, and projected values are summed up to obtain a pixel value [56]:

(1) $$\frac{\delta \sigma }{\sigma }=B\frac{\delta V}{V}$$

where *V* is voltage, *σ* is conductivity values, and *B* ∈ *R^N X LK^*, where *K* is the number of current patterns, *L* is the number of electrodes, and *N* is the number of parameters to be estimated. The normalized changes in the electrical conductivities can be computed as

(2) $$\frac{\delta \sigma }{\sigma }={\left(J_{n}B\right)}^{-1}B\frac{\delta V}{V}$$

where forward operator

*J_n_* = diag (*V*_1_^1^, *V*_2_^1^, *V*_3_^1^, …, *V*_L_^K^)*J*, (3)

The Gauss–Newton method is used as a standard inverse algorithm to solve static measurements in the ERT [57]. The objective function $\varphi \left(\rho \right)$ is formulated to minimize the error in the least square sense,

(4) $$\varphi \left(\rho \right)=\frac{1}{2}\left\{∥U\left(\rho \right)-V{∥}^{2}\;+\alpha \;∥R_{\rho }{∥}^{2}\right\}$$

where $U\left(\rho \right)$ are the voltages calculated through finite element formulation, *V* is measured voltages, $R_{\rho }$ is a matrix, and α is a regularization parameter.

The total variation of a conductivity image is defined as [58],

(5) $$TV\left(\sigma \right)=\int_{\Omega }^{}\left|\left(\nabla \sigma \right)\right|d\Omega$$

where σ is the conductivity vector, and Ω is the region to be imaged. In static image reconstructions, the aim is to obtain the conductivity of the region under analysis. The reconstruction is stabilized using a regularization parameter in the equation, where forward operator *F* and conductivity vector are related, as

(6) $$V=F\;\left(\sigma \right)$$

(7) $$\sigma_{rec}=\arg\;\min\frac{1}{2}∥F\;\left(\sigma \right)-V_{meas}{∥}^{2}+\;\alpha G\left(\sigma \right)$$

where $V_{meas}$ is the vector of the measured voltages, $F\;\left(\sigma \right)$ is the forward model prediction, $G\left(\sigma \right)$ is the regularization functional, α is the hyperparameter which controls the level of the applied regularization, and ∥.∥ is the 2-norm. The total variation functional has an important role in regularization of the inverse problems [59]. The TV functional is advantageous, as it preserves the discontinuities between the phases.

These algorithms were implemented using EIDORS v3.10 [51,52,53]. MATLAB version 2019b was used to obtain the reconstructed images. EIDORS is a software project to provide algorithms implemented in MATLAB/Octave for forward and inverse modeling for electrical resistance tomography. 

### 2.3. ERT and Quantitative Spatial Evaluations

**Figure 3:**
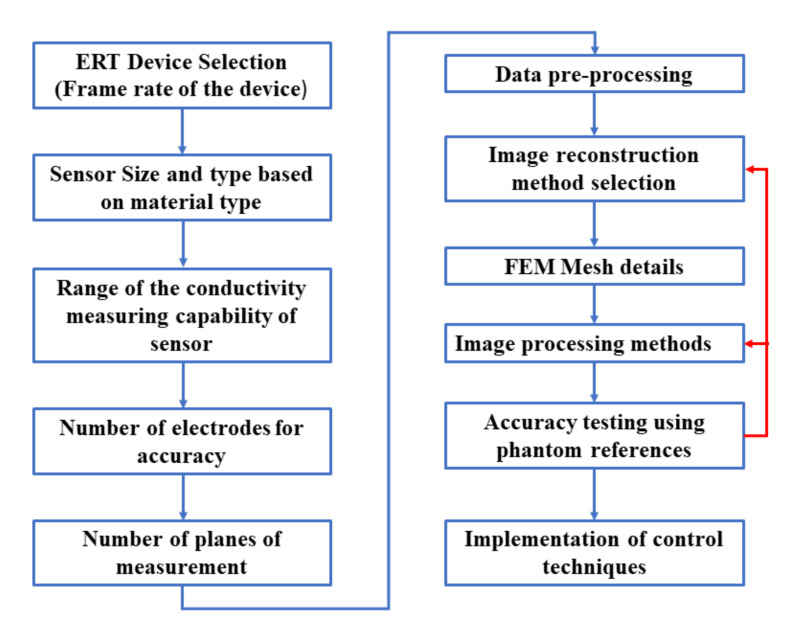
Factors affecting quantitative measurements using ERT as an imaging modality for the crystallization process.

Geometric evaluations in hard-field tomography, such as industrial X-ray computed tomography, are done by using ruby spheres and the concept of radial pairs to evaluate accuracies and errors in the reconstructed images [60]. In soft-field tomography, such as ERT, many factors influence the quantitative accuracy in the spatial domain for the reconstructed images. Some of these factors are limited by the data acquisition system, and others due to various choices made in the data processing stages. Several factors according to which an engineer or an analyst can make a prior decision for a targeted quantitative evaluation using the ERT device are mentioned in Figure 3. For example, the methodology for the data acquisition can either be a V-C or a C-V [54]. A higher frame rate is required for the process technology involving a reaction crystallization, compared to the cooling crystallization. The contact impedance is determined by the size of the electrode, the material of the electrode, and its noncorrosive properties in the industrial environments.

The number of electrodes determines the resolution of the reconstructed image. A higher number of electrodes provide a better resolution in the reconstructed image [61]. The analog to digital converter used determines the voltage/current resolution, which in turn determines the applicability of the data acquisition unit in the low-conductivity environments. The FEM mesh density decides the smallest spatial unit in the image and also is a limiting factor for the resolution. The reconstruction type utilized, either deterministic or probabilistic, affects the proper visualization of data. A review of reconstruction methods for ERT imaging modality was studied [56,62,63,64]. In the case of iterative algorithms, the time taken for the evaluations increases with the number of iterations required. The further morphological or noise removal image processing methods, such as erosion or dilation, are determined by the object and SNR of the image. Once the spatial accuracies are correctly determined, then, based on this information, control and monitoring can be implemented within a closed loop. To achieve quantitative accuracy for the purpose of implementation of the control loop for crystallization monitoring and data processing steps, phantoms of standardized sizes have to be tested in static mode at central and noncentral locations. It is also essential to observe the separability of the objects within the region of interest.

ERT hardware design and testing is an active field of research. The ERT machines for 3D data acquisition are regularly tested for speed and accuracy [65]. High-speed ERT and ECT systems using parallel computing on multi-GPU in a heterogeneous system [66], FPGA electronics [13,67], with parallel and multiplanar ERT systems [68], and rapid estimation algorithms using artificial neural networks [69] have been designed to evaluate various process tomographic parameters.

Quantitative evaluation of small regions in low-conductivity solutions using standard 3D-printed cylindrical phantoms in a 2D plane is the primary objective of this study. This is achieved by comparing the region of the reconstructed phantom image to the corresponding cross-section area of the phantom inside the beaker. Assessment of the overall factors impacting these evaluations would benefit us in quantifying the natural crystallization processes and determine the spread of crystal formations or presence. Some works in this direction are briefly mentioned here. Previous experiments by [46] compared ERT simulations with experiments using the phantoms of diameter 2 cm in a tank of diameter 2.4 m by measuring the differential voltages. The regularization parameters were varied in [58] to evaluate and improve the spatial resolution in separating the simulated phantom objects of different shapes. A novel projection error propagation (PEPR) based regularization parameter was proposed to improve the image quality of the reconstructed image [70]. Image fusion techniques were used to enhance the ERT reconstruction in the simulated images [71]. Quantitative measurements of the simulated phantoms and synthetic data were also conducted by using LBP, Landweber, and Tikhonov reconstruction [72]. However, the accuracy of the real phantoms was not tested in this work quantitatively. Experiments with phantom of diameter 8 cm and iterative Gauss–Newton reconstruction using adaptive mesh in a saline solution was done by [73] without detailed quantitative evaluations. Advanced segmentation methods, such as fuzzy clustering of the data, were implemented on the LBP-reconstructed image separating three phantoms [74]. Also, K-means-based classification of the images acquired using the ERT methods was performed by [75]. These studies did not involve low-conductivity media and accuracy evaluations. They focused on different factors affecting accuracy within their experiments inside solutions.

The overall objective of this work was to test the accuracy of the newly-developed sensor of the ERT system for the accurate representation of nonconductive objects in the low-conductivity solutions, and also to check the influence of selected factors for quantitative measurements. The aim of this work was also to estimate limits of ERT measurement by determining a correlative percentage of the area of the predetermined standard phantoms detectable using ERT in various solutions with different conductivities. These experiments were performed within the framework of the European Union Horizon 2020 TOMOCON project (smart tomographic sensors for advanced industrial process control) [76]. The focus of the TOMOCON project is to create a multi-sensor network to monitor, visualize, and control batch crystallization processes.

## 3. Experimental Design

The summary of the conducted experiments is shown in Table 1.

### 3.1. Experimental Setup and Sensor Design

The experiment was conducted using the laboratory-based batch reactor with an internal diameter of 83 mm. Sixteen equidistant and circular surfaced electrodes were placed to acquire the 2D static image. The electrodes were made of stainless steel. They were inserted after punching holes in the plastic reactor. Rubber washers were used to prevent the leakage of the solutions from the inside of the reactor towards the outer environment. The distance between the electrodes was 5.19 mm. The diameter of the electrode-head screw was 12 mm. The total surface area in contact with the medium for each electrode was 113 mm^2^. Reactor measurement sizes are shown in Table 2.

**Figure 4:**
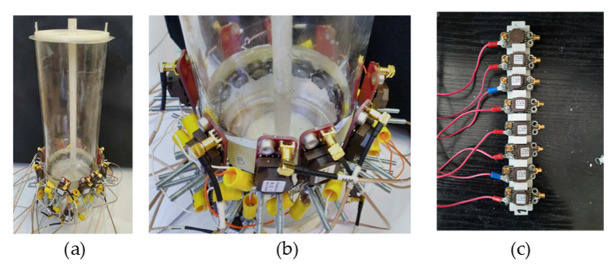
(**a**,**b**) Setup of the laboratory-based batch reactor with sensor and signal conditioning unit mounted on the reactor; (**c**) signal conditioning unit mounted on the 3D-printed frame.

An ERT system from Rocsole Ltd. was used for data acquisition and processing. Figure 4a,b show the reactor with a mounted sensor [36]. All the signal conditioning units were mounted on the signal conditioning unit holder, as shown in Figure 4c. The new signal conditioning unit was connected to electrodes with a specially insulated coaxial cable, type RG178, of length 2.5 m. The RG178 coaxial cable measuring 2.5 m was used to connect the signal conditioning unit to the ERT data acquisition FPGA of the Rocsole system. This sensor and signal conditioning unit was used in order to perform experiments in the low-conductivity solutions. It contained transformer coils with F-inductives at the value of 9/3:1. The MCX connectors were used to connect the sensor output to the data acquisition system (DAS) of the Rocsole device. The raw data of the static evaluations, to obtain the results, was retrieved from the memory stored by the Rocsole device for evaluations.

Tap water, industrial-grade saturated sucrose solution, and demineralized water measuring 250 mL were used for the experiment. The mass fraction of the sucrose in the industrial-grade saturated sucrose solution was 66.67% *w*/*w*. This volume was constant across all the experiments. The room temperature, as well as the temperature of the solution, during the experiment was 19 degrees Celsius. The conductivity of the tap water and demineralized water was evaluated at 60.1 mS/m and 10.1 mS/m, respectively. For image reconstructions using Gauss–Newton algorithm and total variation algorithm, the Jacobian background value was set to 1.

The voltage–current (VC) evaluation methodology was utilized [54]. In this type of methodology, implemented using 16 electrodes, E1 to E16, the evaluation is done in the following manner. The E1 (source electrode) is excited using voltage, and the remaining electrodes (sink electrodes) simultaneously acquire the currents. This acquisition process is continuously repeated for all the other electrodes. The source electrodes are sequentially changed from E2, E3, until E16, and data frames are recorded. The data was acquired by the data acquisition system FPGA at an average frame rate of 14 Hz. The data was transferred via a local area network (LAN) connection to memory. The stored memory data was utilized for the reconstruction of the static images.

### 3.2. Phantom Design and 3D Printing

A total of six phantoms were evaluated, as shown in Table 3. Five cylindrical phantoms with the decreasing diameter were 50 mm, 40 mm, 30 mm, 20 mm, and 10 mm. The sixth phantom was 10 mm × 2 with a distance of 45 mm between their centers. The expected area percentages of phantoms in 2D reconstructions is also shown in Table 3. Phantom R6 was tested at two locations, L1 and L2. The phantoms were 3D-printed using acrylonitrile butadiene styrene (ABS) material in-house, using a 3D printer at the Lodz University of Technology. The phantoms were designed using the software Blender, version 2.79b. An Ultimaker-3 Extended 3D printer device was used to print the phantoms, with the help of Ultimaker Cura 4.6 software. The phantoms were completely filled with ABS, and no hollow space was left inside the structure. The property of the ABS is that it is electrically insulating in nature.

**Figure 5:**
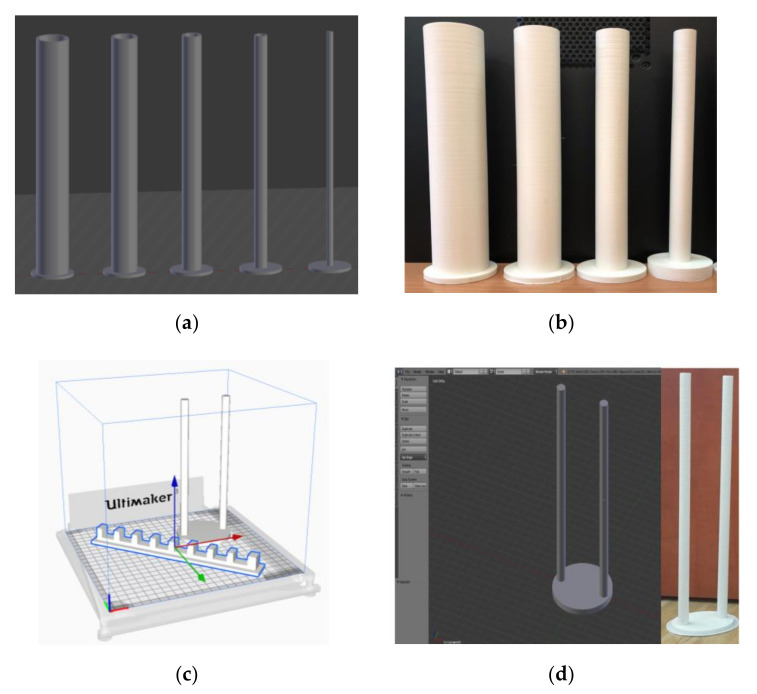
(**a**) Design of phantoms R1–R5; (**b**) 3D-printed acrylonitrile butadiene styrene (ABS) phantoms; (**c**) printing of sensor mounting unit and phantom R6; (**d**) design and print of phantom R6.

The phantoms were relatively heavy, compared to the polylactic acid (PLA) phantoms used previously [36]. They were stable in the liquid and did not fall due to the buoyant force exerted by the liquids. Figure 5a shows the 3D phantoms R1 to R5, designed using Blender v 2.79 software. Figure 5b shows the printed phantoms. Figure 5c shows phantom R6 and signal conditioning unit holder in Cura GUI to be printed. Figure 5d displays the design of phantom R6 and 3D-printed ABS phantom.

### 3.3. Sucrose Crystal Agglomerate Assesments, Experimental Progression, and Test Objectives

The sugar crystals do not conduct electricity. Saturated sugar solution and its crystallization and granulation have been studied as a subject of human and animal nutrition. The scientific study for its solubility properties and crystallization properties was carried out by [77]. The solubility of sucrose in water, supersaturation, and sucrose crystal growth are essential physical properties to be understood for the implementation of PAT in the batch crystallization process [78]. Sugar forms a covalent bond with water, and sucrose molecules remain intact and do not dissociate in the same manner as ionic compounds. Hence, the decrease in electrical conductivity is observed during the crystal growth process. This poses several challenges for the evaluations during crystallization within a reactor, for instance, simultaneous precipitation and conductivity changes in the electrode plane. It is essential for the implementation of the control that the reconstruction methods provide morphology of the phantoms, which correlates to the physical sizes of crystals at various locations within the reactor [36]. One of the challenging tasks we are involved with for the evaluation of sucrose crystallization is growing crystals at a specific location. Using controlled experiments, the crystal growth can be achieved over a cold cylindrical object at the center of the beaker. Using standard cylindrical phantoms, the crystal growth over the cold test object can be evaluated.

The progression of the experiment was as follows. Electrodes made of stainless steel were used. The new signal conditioning unit was specifically designed, with the help of Rocsole Ltd., for measurements in the very low-conductivity solutions. Phantoms were designed and 3D-printed. The industrial-grade sucrose solution was obtained from the Polski Cuckier company. A total of 720 frames of data were acquired and averaged to do the processing for every static phantom at the center. Phantom R6 was evaluated in two positions, located at L1 and L2.

All images were reconstructed using three reconstruction algorithms on the FEM model type “f2c” with 2304 elements, as shown in Figure 2b. The results for solutions with different conductivity are shown in Section 4.1. For the total variation method, the number of iterations was varied from 2 to 12. Thereafter, the obtained reconstructed images were segmented using Otsu segmentation, local adaptive threshold, and K-means segmentations. Additionally, three color channels were separated, and images are visualized in Section 4.3. The influence of changes in iterations using contrast level mapping for the green channel is discussed in Section 4.4 and presented in Appendix A. The green channel with 256 levels was extracted and binarized using various thresholds ranging from 0.1 to 0.9. A combination of the influence of the number of iterations, threshold levels for green channel binarization, and morphological processing method erosion is analyzed and presented in Section 4.4. In Section 4.5, application of the analyses is tested for real-time monitoring using sucrose crystals in demineralized water.

## 4. Results

This section shows the results of the experiments performed and progresses in the following manner.

### 4.1. Differences Due to Reconstruction Methods

**Figure 6:**
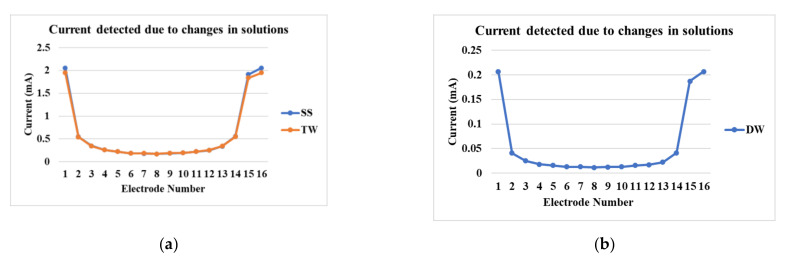
Current detection at various electrodes in the reactor for (**a**) industrial-grade saturated sucrose solution and tap water, and (**b**) demineralized water.

Figure 6a shows the currents detected at various electrodes for tap water and industrial-grade saturated sucrose solution. The detected currents are in the range from 0.25 mA to 2 mA. The differences between the currents detected for tap water and the industrial-grade saturated solution is minimal due to the use of tap water for sucrose production processes, whereas in Figure 6b, the demineralized water has the current range from 0.025 mA to 0.2 mA. It is clear from these results that our new signal conditioning unit is able to distinguish the small current magnitudes in the low-conductivity solution.

**Figure 7:**
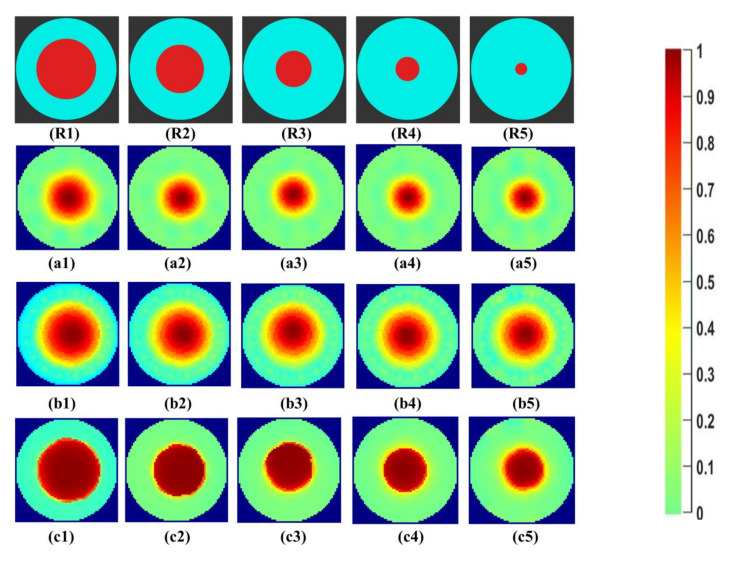
(**R1**–**R5**) phantom reference; (**a1**–**a5**) Gauss–Newton (GN) reconstructions; (**b1**–**b5**) linear back projection (LBP) reconstructions; and (**c1**–**c5**) total variation (TV) reconstructions at 10 iterations for tap water.

Initial qualitative analysis of the reconstructed images for the tap water solution for the phantoms R1 to R5 can be seen in Figure 7. The expected percentage area of phantoms inside the reactor, calculated from Equation (8), is given in Table 3. These values are ideal reference values calculated for phantoms with a circular cross-section inside the reactor. The first row, R1–R5, shows the reference images of the phantoms created using the CAD software Blender v 2.79. The areas of the phantoms and the beaker were designed with ideal diameters to be compared with the reconstructed images. The second row, a1–a5, shows the Gauss–Newton reconstructions for the image. In b1–b5, the reconstructions for the linear back projection method can be seen. In the last row, c1–c5 reconstructions using iterative total variations with ten iterations are presented. The number of iterations was set to ten for comparison purposes with other reconstruction methods. The lesser number of iterations did not provide a sharp boundary, as further studied in Section 4.2. 

All the reconstruction methods successfully reconstruct the objects at the center for the phantoms R1 to R5. Visible qualitative differences are observed in the reconstructions of the same object using different algorithms. For the Gauss–Newton method, a growing trend in size can be seen with a smooth change in color space at the boundary regions. For LBP reconstructions, the reconstructed area is overestimated for all sizes. Total variation provides sharp edges to determine the boundary of the central object. The color bar from 0 to 1 correlates to the region with the least and highest conductivity within the image for the background calculated for the difference evaluations.

The area of the phantom for quantitative analysis is calculated using Equation (8):

(8) $$Percentage\;area\;of\;phantom\;(A_{P})=\frac{Number\;of\;pixels\;with\;segmented\;phantom\;region\;}{Number\;of\;pixels\;representing\;reactor\;region}\times 100$$

**Figure 8:**
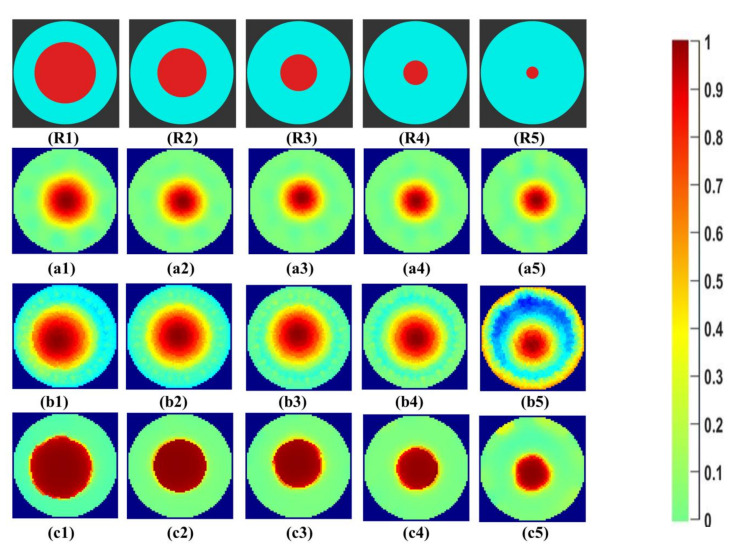
(**R1**–**R5**) phantom reference; (**a1**–**a5**) Gauss–Newton reconstructions; (**b1**–**b5**) LBP reconstructions; and (**c1**–**c5**) TV reconstructions at 10 iterations for industrial grade saturated sucrose solution.

Initial qualitative results of the reconstructed images for the industrial-grade saturated sucrose solution for the phantoms R1 to R5 can be seen in Figure 8. The first row, R1–R5, shows the reference area represented by the phantoms. The second row, a1–a5, shows the Gauss–Newton reconstructions for the image. In b1–b5, the reconstructions for the linear back projection method can be seen. In the last row, c1–c5, reconstructions using iterative total variations can be seen for the ten iterations.

The Gauss–Newton method and total variation method successfully reconstructs the phantoms R1 to R5. LBP reconstructions have significant noise in the background of the image. Higher image artifacts are seen for the R5 phantom with LBP reconstruction. The overestimation for the size of the phantoms R3 to R5 can be visualized.

**Figure 9:**
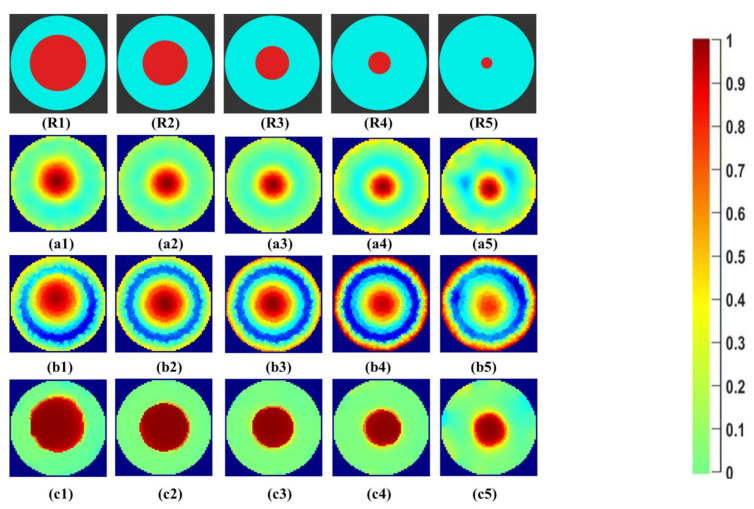
(**R1**–**R5**) phantom reference; (**a1**–**a5**) Gauss–Newton reconstructions; (**b1**–**b5**) LBP reconstructions; and (**c1**–**c5**) TV reconstructions at 10 iterations for demineralized water.

Initial qualitative results of the reconstructed images for the demineralized water with the lowest conductivity in our experiments for the phantoms R1 to R5 can be seen in Figure 9. The first row, R1–R5, shows the reference area represented by the phantoms. The second row, a1–a5 shows the Gauss–Newton reconstructions for the image. In b1–b5, the reconstructions for the linear back projection method can be seen. In the last row, c1–c5, reconstructions using iterative total variations can be seen for the ten iterations.

The Gauss–Newton method reconstructs the phantoms successfully, but for the phantom R5, the background noise can be observed, which indicates lower SNR. LBP reconstructions fail to resolve the background and objects. This would require a significant image processing effort to separate the central object. The total variation method with ten iterations provides a clear separation between object and background. Phantom R3 to R5 appears to be over-estimated in size. Segmentation and binarization lead us to quantitative values of the nonconductive region, which are discussed in Section 4.3 and Section 4.4. In further results, only demineralized water is analyzed for size and separability as this is the most challenging case, and relevant for practical applications of crystal growth using antisolvent techniques.

**Figure 10:**
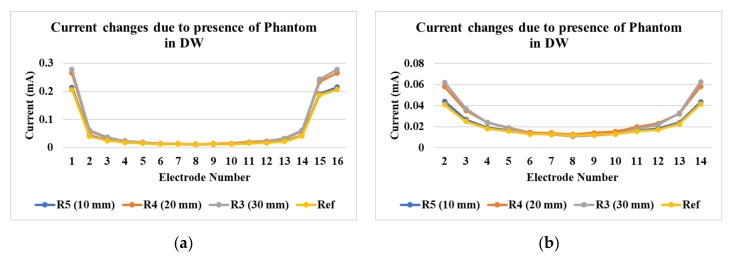
(**a**) Changes in current at various electrodes after phantom placement; (**b**) detailed view from electrode 2 to 14.

**Figure 11:**
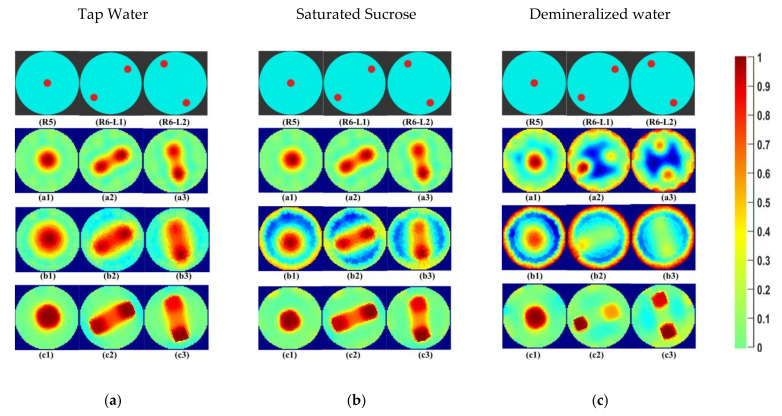
(**a**) Tap water; (**b**) industrial-grade saturated sucrose solution; (**c**) demineralized water: (R5, R6-L1, R6-L2) phantom reference; (**a1**–**a3**) Gauss–Newton reconstructions; (**b1**–**b3**) LBP reconstructions; (**c1**–**c3**) TV reconstructions at 10 iterations.

In Figure 10a, the differences in the currents acquired for the demineralized water with phantoms R3, R4, and R5 are shown. Figure 10b shows differences at electrode numbers 2 to 14. It can be seen that the maximum absolute difference between currents detected at electrode 2 is 0.02 mA. In Figure 11, reconstructions of phantom R5 and R6 at locations L1 and L2 can be seen qualitatively. In Figure 11a, the differences between the reconstructions for tap water are visible for phantom R6. The results are similar to phantoms R1–R5 in Figure 7. In Figure 11b, for industrial-grade saturated sucrose solution, the objects are separated only in total variation segmentation. Other methods give background noises. The LBP method in Figure 11c fails to detect and separate phantoms in demineralized water. The phantoms are separable and visible with the total variation method.

### 4.2. Varying the Iterations in the TV Reconstruction

**Figure 12:**
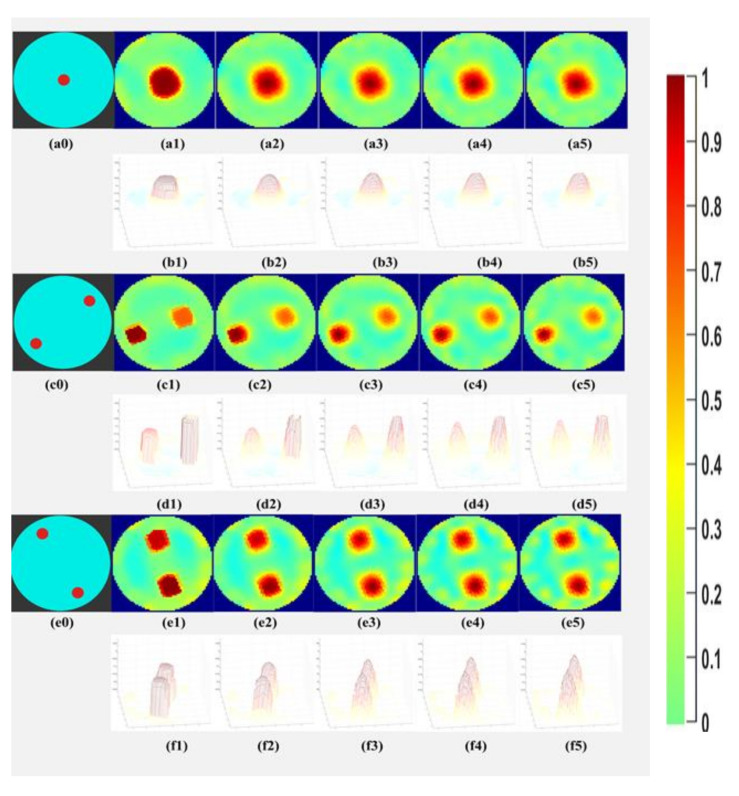
(**a0**–**a5**) phantom R5 reference and TV iteration 10,8,6,4,2; (**b1**–**b5**) surface plot of the reconstructed images for phantom R5; (**c0**–**c5**) phantom R6-L1 reference and TV iteration 10,8,6,4,2; (**d1**–**d5**) surface plot of the reconstructed images for phantom R6-L1; (**e0**–**e5**) phantom R6-L2 reference and TV iteration 10,8,6,4,2; (**f1**–**f5**) surface plot of the reconstructed images for phantom R6-L2.

The tests were continued with the case demineralized water, phantom R5, R6-L1, and R6-L2 to determine if the object detection and separability could be obtained at lower iterations. In Figure 12, the object separability is achieved for the demineralized water, even if the number of iterations is lowered until 2. It is also observed that the background noise increases while the number of iterations is reduced. Figure 12 also shows surface plots to signify that as the number of iterations decreases, the change in the contrast of the detected object boundary is gradual and not sharp. These results motivated us to segment the reconstructions with the least iterations and to observe the area covered quantitatively for all the phantoms in our further analyses.

### 4.3. Analysing Segmentation Methods and Morphological Image Processing

**Figure 13:**
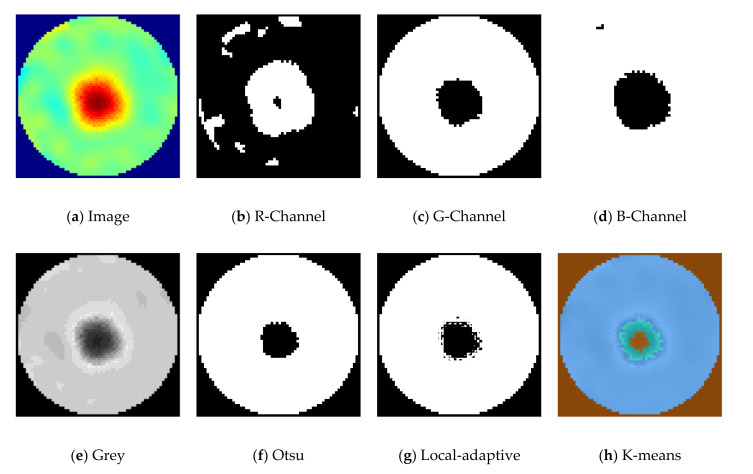
Various image segmentation methods for phantom R5. Solution: demineralized water, reconstruction method: total variation, iterations: 2.

The extracted RGB color channels for phantom R5 in demineralized water are shown in Figure 13b–d. Additionally, image segmentation using Otsu, local-adaptive threshold, and the K-means method with three regions for phantom R5 is shown in Figure 13f–h. They were obtained by using the MATLAB image processing toolbox functions otsuthresh(), adaptthresh(), and imsegkmeans(). The local adaptive threshold creates multiple nonconnected regions in the image. This would require additional processing methods. With the K-means method evaluating three regions, the boundary region of the phantom R5 is detected as a separate region. The over-segmentations using Otsu and G-Channel segmentation methods for phantom R5 are visualized in Figure 13c,f, respectively.

**Figure 14:**
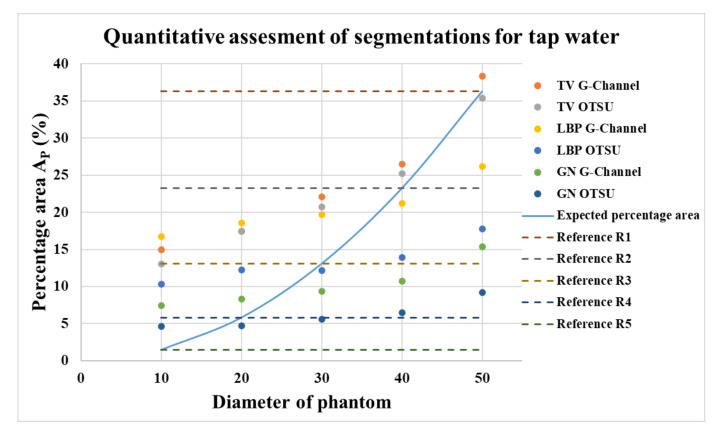
Comparison of the area percentage for Otsu and G-Channel segmentation using TV, LBP, and GN reconstructions for (a) R1, (b) R2, (c) R3, (d) R4, and (e) R5.

The quantitative assessments for segmentation using the G-Channel and Otsu method for tap water with the reconstruction algorithms TV, LBP, and GN are shown in Figure 14. The correlations for these percentage areas of phantom regions (A_P_) with phantom diameters (PD) and the expected percentage area of phantoms are shown in Table A1 in the Appendix A. The effect of applying erosion as morphological processing to obtain is shown in Figure A2a–e in the Appendix A. The E0 to E30 signifies the morphological image processing erosion applied. E0 stands for no erosion applied, and E10, E20, and E30 stands for incremental erosion applied using ‘disk’ operation in MATLAB image processing toolbox with 10, 20, and 30 as the radius, respectively. 

It can be observed in Figure 14 that the GN algorithm shows promising results for the smaller phantoms R4 and R5, but under-segments for the larger phantoms R1 to R3. Similarly, phantoms R1 to R3 are under-segmented using the LBP algorithm, and R4 to R5 are over-segmented using both segmentation methods. Phantoms were not reconstructed or segmented in demineralized water (low-conductivity solution) using the LBP algorithm. For the TV algorithm with ten iterations, the results were closest to the expected reference area percentage for the phantoms R1 to R3. Phantoms R4 and R5 were over-segmented. The standard deviations shown in Table A1 presents the need for further analyses for phantom R4 and R5.

**Figure 15:**
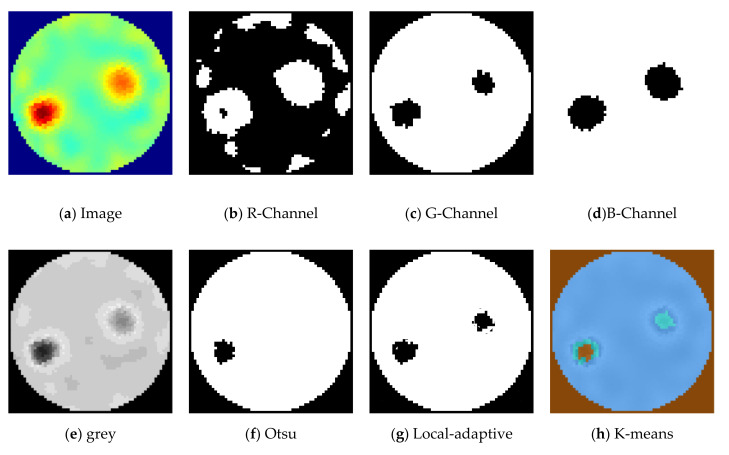
Various image segmentation methods for R6-L1. Reconstruction method: total variation, iterations: 2, solution: demineralized water.

The extracted RGB color channels for phantom R6-L1 (location 1) in demineralized water are shown in Figure 15b–d. Additionally, image segmentation using Otsu, local-adaptive threshold, and the K-means method with three regions for phantom R6-L1 can be seen in Figure 15f–h. Otsu segmentation fails to detect the second phantom. With G-Channel segmentation, both the phantoms are visible with the binarization threshold set at 0.6. The binarization threshold can be varied from 0.1 to 0.9 and is used after extracting the green color channel (G-Channel) image.

The results from the evaluations presented from Section 4.1 and Section 4.2, Section 4.3 help us to reach the factors that are best suited as the initial settings for the evaluations in low-conductivity solutions. They are; reconstruction method: TV, segmentation method: G-Channel, threshold level: 0.6, and erosion: E0. The dependency of the parameters and their effect on the obtained percentage area as the size of the phantoms decrease from 50 mm (R1) to 10 mm (R5) are analyzed in Section 4.4.

### 4.4. Towards Quantitative Estimations Using a Combination of Image Processing Methods to Achieve the Expected Area Estimation

**Figure 16:**
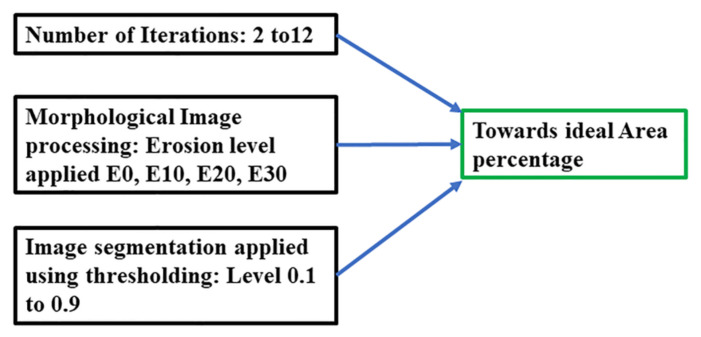
Combination of image processing factors to achieve the expected percentage accuracy.

Three parameters can be varied to achieve the percentage area for the phantom as targeted for the ideal circle, as presented in Table 3. The number of iterations (2–12), image segmentation threshold (0.1 to 0.9) for G-Channel segmentations, and morphological image processing parameters (E10 to E30) in case of over-segmentation are summarized in Figure 16.

#### 4.4.1. Contrast Profile Assessment at Various Iteration Levels

**Figure 17:**
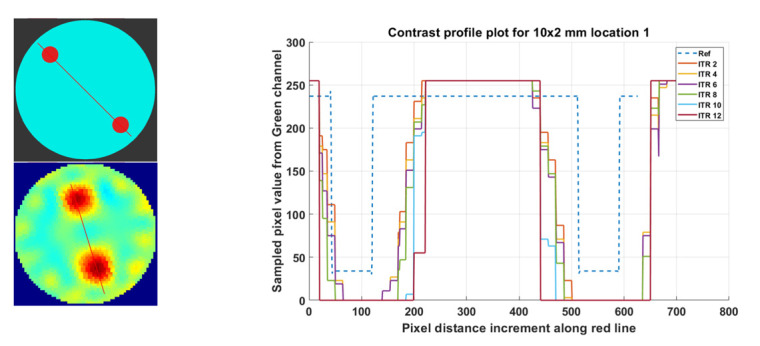
Contrast profile plot for phantom R6-L2 (2 × 10 mm), reconstruction: TV, iterations: 2–12; channel: green, location: 2.

**Figure A1:**
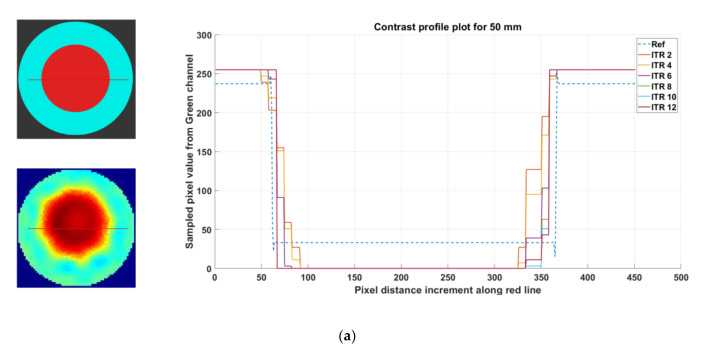

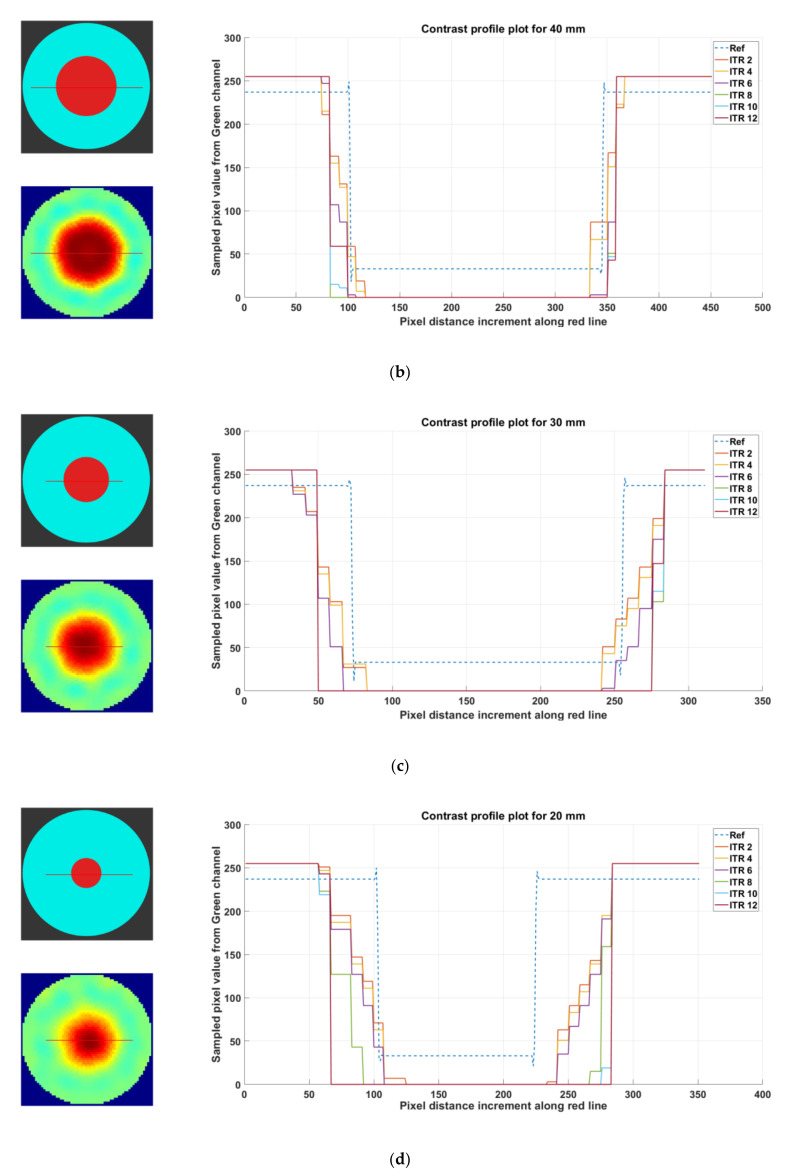

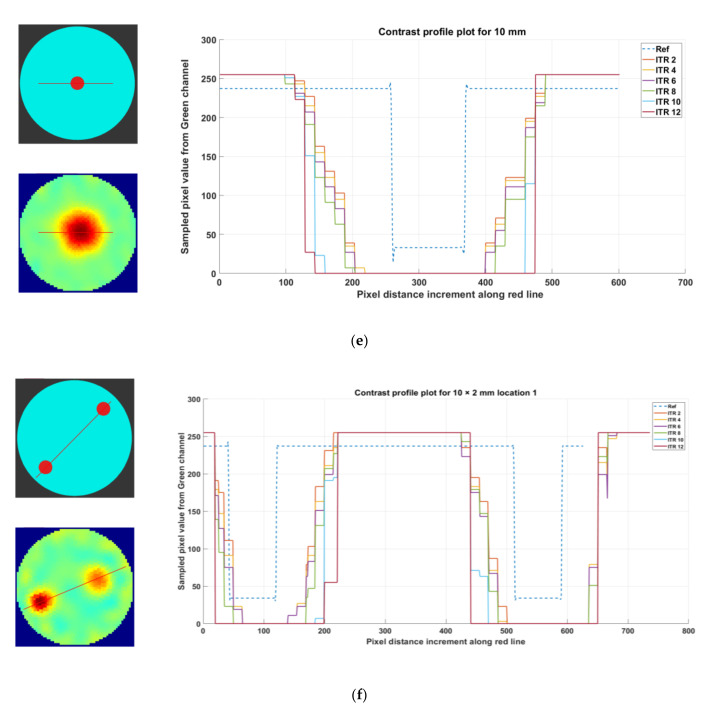
(**a**) Contrast profile plot for 50 mm phantom R1, reconstruction: TV, iterations: 2–12; channel: green. (**b**) Contrast profile plot for 40 mm phantom R2, reconstruction: TV, iterations: 2–12; channel: green. (**c**) Contrast profile plot for 30 mm phantom R3, reconstruction: TV, iterations: 2–12; channel: green. (**d**) Contrast profile plot for 20 mm phantom R4, reconstruction: TV, iterations: 2–12; channel: green. (**e**) Contrast profile plot for 10 mm phantom R5, reconstruction: TV, iterations: 2–12; channel: green. (**f**) Contrast profile plot for phantom R6-L1 (2 × 10 mm), reconstruction: TV, iterations: 2–12; channel: green, location: 1.

**Figure A2:**
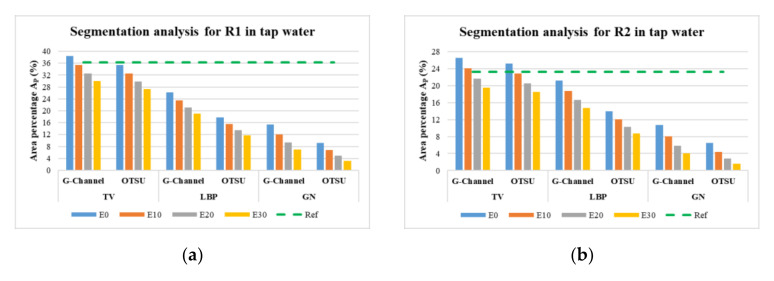

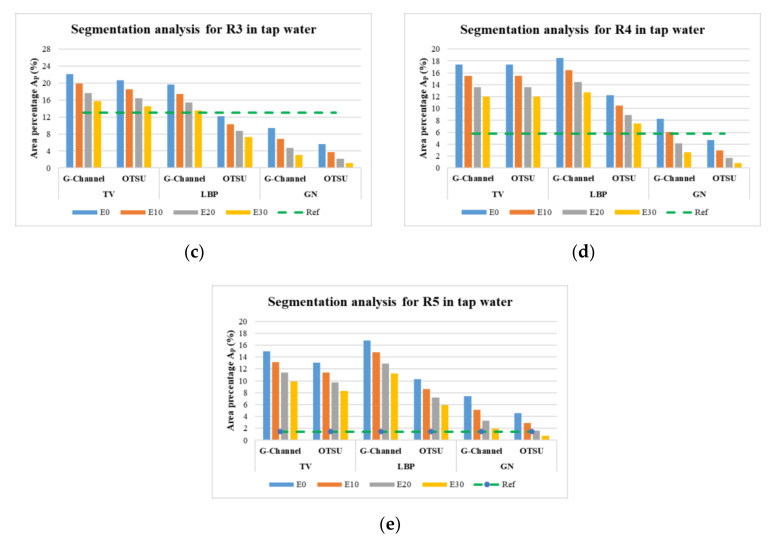
Comparison of the area percentage for Otsu and G-Channel segmentation using TV, LBP, and GN reconstruct (**a**) R1 (**b**) R2, (**c**) R3, (**d**) R4, and (**e**) R5.

The influence of the change in iterations using contrast profile plots for phantoms R1 to R5 on the G-Channel segmentation is shown in Appendix A Figure A1a–g. In Figure 17, the variation in iterations for the phantoms R6-L2, using contrast profile plot for the G-Channel segmented with threshold 0.6, is shown in Figure 16a–g. The contrast profile plots are compared to the reference images. It can be observed that lower iterations give results near to the actual width of the reference phantoms.

#### 4.4.2. Evaluation of the Area Covered by Phantoms at Various Iterations

**Figure 18:**
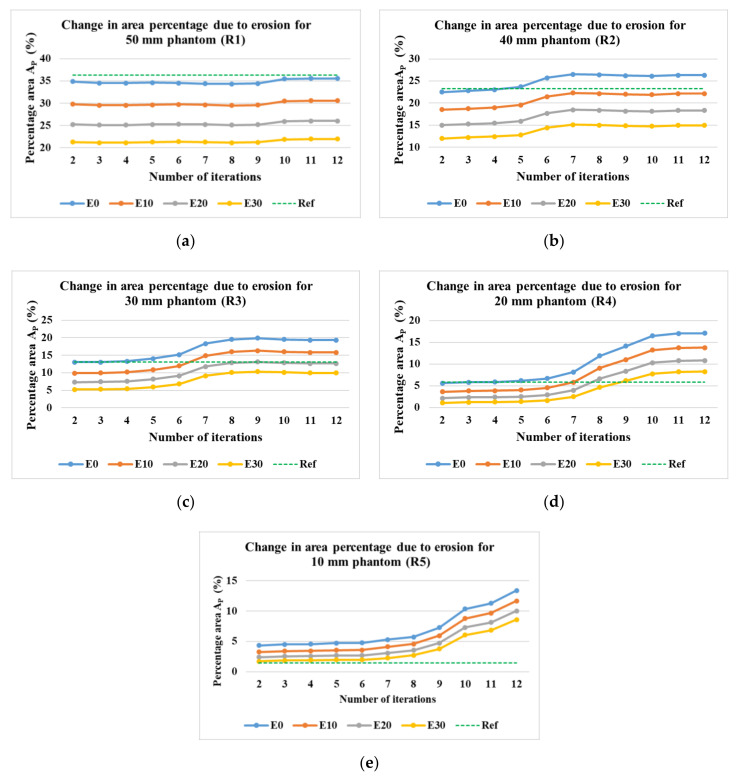
Percentage area covered by phantoms at constant imaging threshold level and the varying number of iterations and erosion factors. Reconstruction: TV, iterations: 2–12; channel: green; for the phantoms (**a**)R1; (**b**) R2; (**c**) R3; (**d**) R4; (**e**) R5.

**Figure 19:**
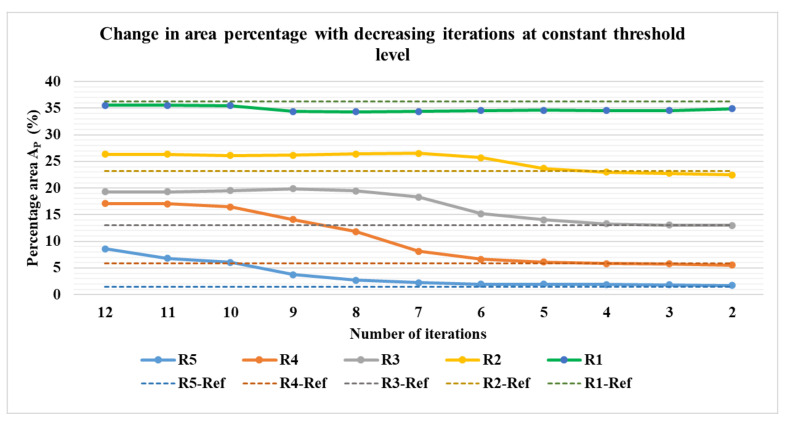
Percentage area covered by phantoms at constant imaging thresholds and erosion factor and a varying number of iterations: combined view. Reconstruction: TV, iterations: 2–12; channel: green.

Figure 18a–e show the percentage of the area covered by the phantoms at various iterations for the phantom R1 to R5. The image processing method of erosion with the help of MATLAB function erode() and strel morphological structuring element disk with the radius of the scale 10 (E10), 20 (E20), and 30 (E30) were applied to the images to evaluate the percentage area of the phantoms from the image. The different threshold levels were set for the evaluation of the data 0.9 for R1, 0.2 for R2, 0.3 for R3, 0.1 for R4, and 0 for R5. This was done in order to obtain expected results without application of erosions and at the lowest number of iterations possible. It was observed that for the phantoms R1 to R4, the expected values of the area could be achieved without the application of any erosion at E0. For phantom R5, the expected area was achieved after the application of erosion of E30. It was observed that for phantom R1, the expected percentage value was achieved at higher iterations, whereas, for R2 to R5, it was achieved at lower iterations. It can be observed from Figure 19 that for phantoms R2 to R5, as we reduce the number of iterations the value of the area decreases towards the expected percentage area. A lower number of iterations for reconstruction algorithm also has an added advantage for higher speed of evaluation.

#### 4.4.3. Evaluation of the Area Covered by Phantoms at Various Threshold Levels

**Figure 20:**
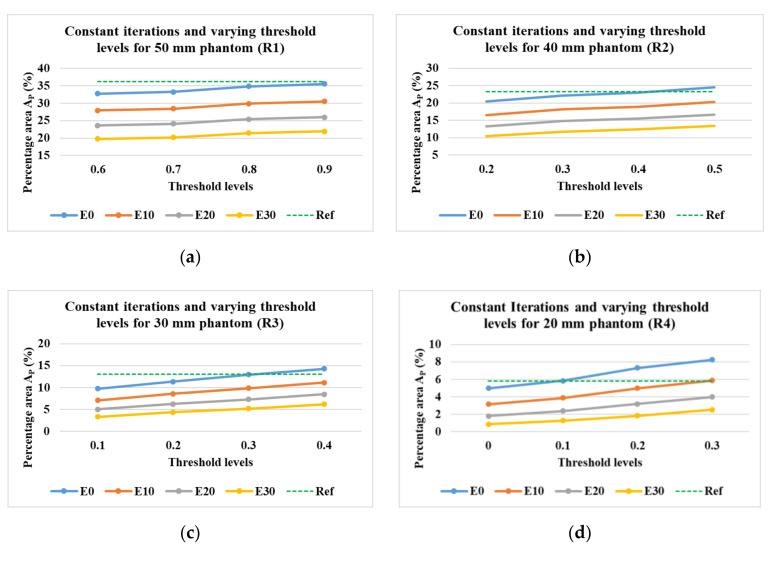

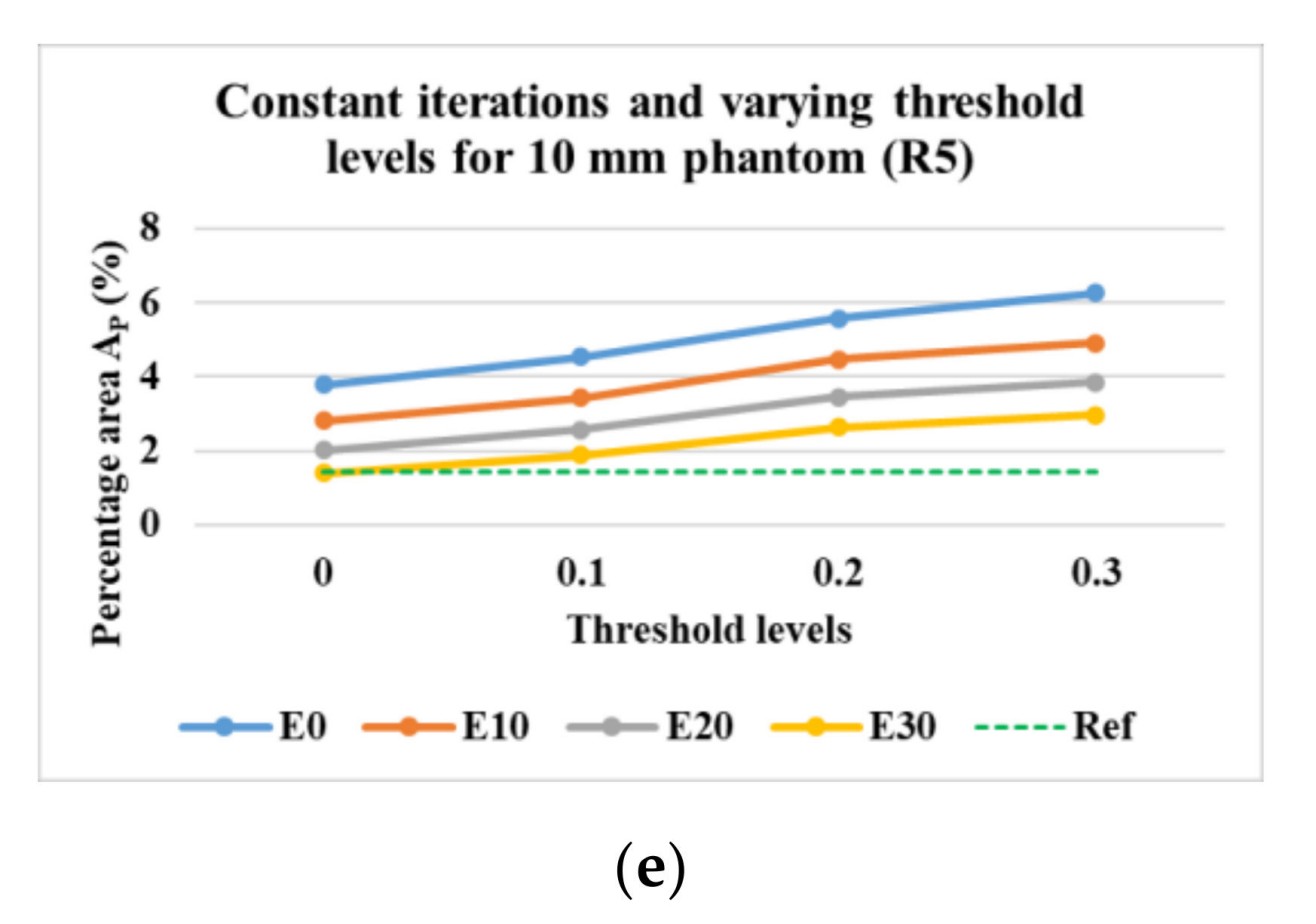
Percentage area covered by phantoms at a constant number of iterations and varying imaging thresholds and erosion levels. Reconstruction: TV, iterations: 2; channel: green; for the phantoms (**a**) R1; (**b**) R2; (**c**) R3; (**d**) R4; (**e**) R5.

It was also explored whether, instead of the application of morphological image processing, a similar result could be obtained by changing the values of the thresholds to binarize the green color channel. The iterations for the evaluations were constant at 2. Figure 20a–e shows the results achieved varying the threshold levels from 0.6 to 0.9 for the phantom R1, 0.2 to 0.5 for R2, 0.1 to 0.4 for R3, 0 to 0.3 for R4, and 0 to 0.3 for R5.

**Figure 21:**
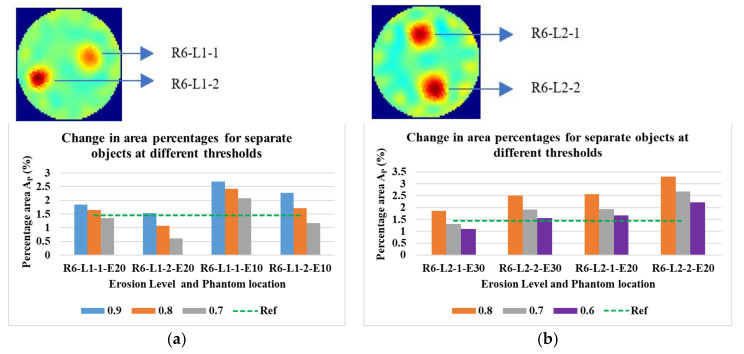
Percentage area covered by 2 × 10 mm (**a**) phantom 1 and (**b**) phantom 2 at various threshold levels. Reconstruction: TV, iterations: 2, channel: green.

Figure 21 shows the phantoms R6-L1 and R6-L2 at two erosion levels and three threshold levels. The individual phantoms are 10 mm in diameter.

**Figure 22:**
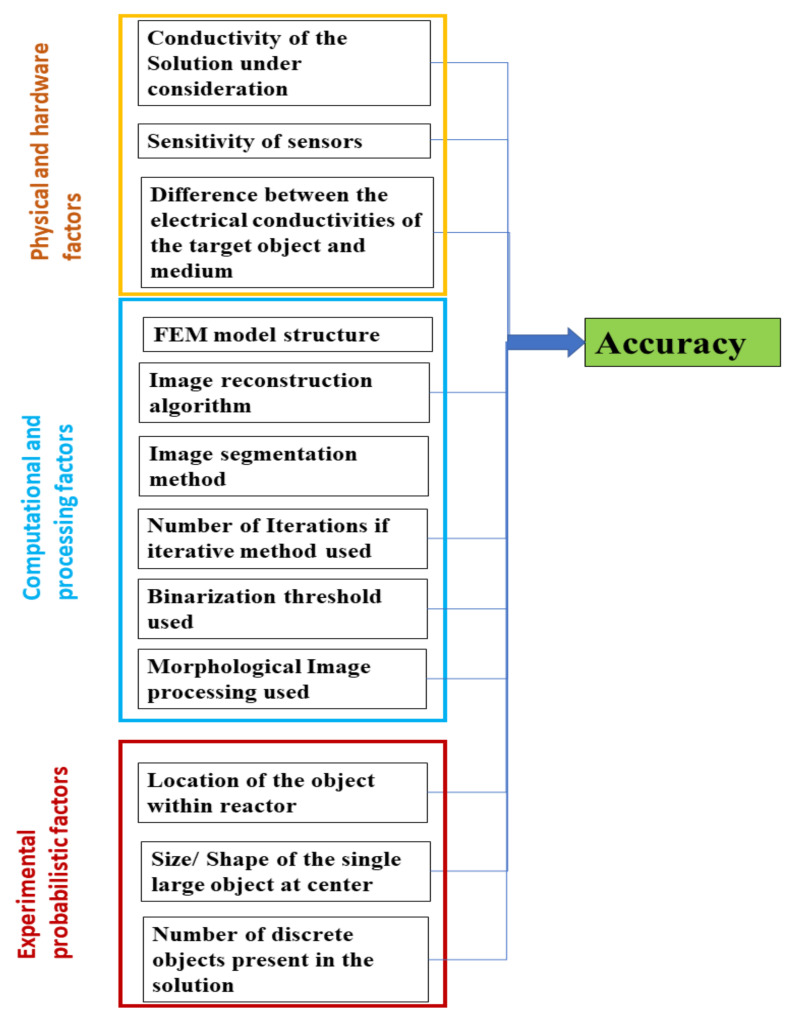
Factors influencing the accuracy of the image object in the ERT-reconstructed image.

Figure 22 mentions a collection of the interdependent factors which affect the accuracy. These can be divided into the factors arising from physical conditions such as differences between conductivities of the solute and solution, hardware, and design. The computational data processing techniques, such as reconstruction algorithm, FEM model structure, the image segmentation method, number of the iterations for the algorithm, and binarization threshold, also play a significant factor in determining spatial accuracy. Additionally, other factors specific to the inverse imaging in soft-field tomographies, such as the distance of the object from the sensor during the experiments, make the assessment of spatial accuracy in ERT very challenging. A standard method for accurate determination of nonconductive objects in such cases hence depends upon multiple variables. A model incorporating these factors and training a neural network for best estimates could offer a potential solution.

### 4.5. Experimental Industrial Application in Assessment of Sucrose Crystals in Demineralized Water

**Figure 23:**
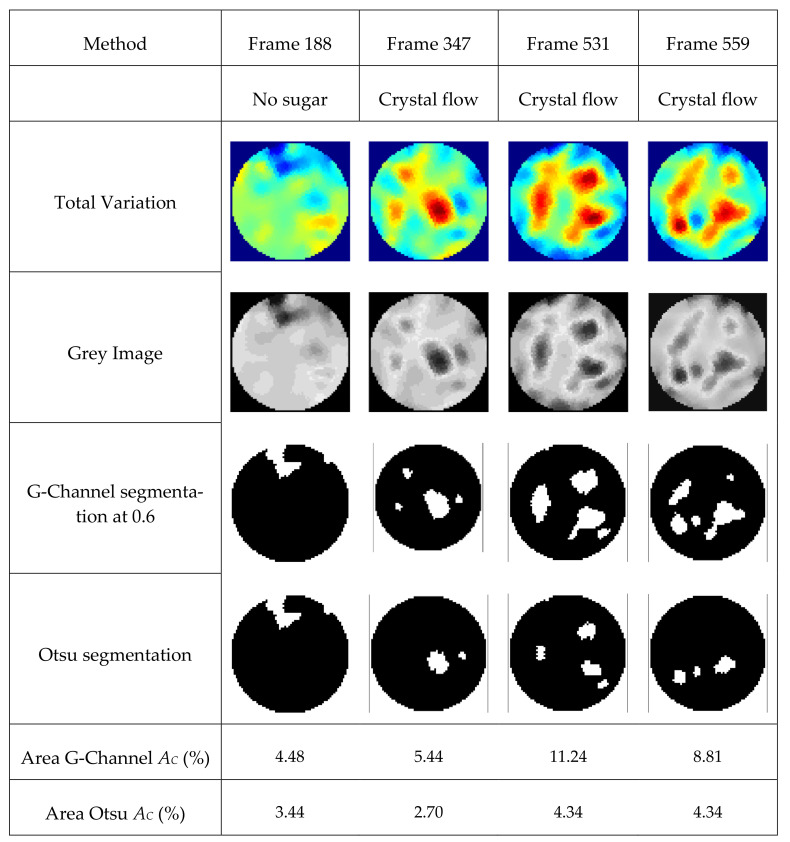
Visualization of the sugar crystals in the demineralized solution at various frames representing time points. Reconstruction: TV, segmentation: Otsu and G-Channel at threshold 0.6.

The practical applications of the reconstructions to the real experiment involving the insertion of sugar in the demineralized water and visualization using a TV reconstruction algorithm is presented in Figure 23. The sugar crystals weighing 250 g were inserted using a funnel into the experimental batch reactor in the central region. The measurements were acquired at the frame rate of 14 Hz. At frame 188, the insertion of sugar was initiated. Figure 23 presents the reconstructed images of the sugar crystals in the demineralized water. The differences in the conductivities are detected. We can see that apparent differences exist in the conductivity profiles. The images were segmented using the Otsu threshold and G-Channel threshold. The area covered by the segmented crystals was evaluated using MATLAB functions bwboundaries and regionprops. Equation (9) evaluates the percentage area of the regions characterizing the crystal regions, *A_C_*. It was observed that the G-Channel segmentation provides better results compared to the Otsu method. The area of the 2D region visualized inside the reactor is compared.

(9) $$Percentage\;area\;of\;crystals\;(A_{C})=\frac{Number\;of\;pixels\;with\;segmented\;region\;}{Number\;of\;pixels\;representing\;reactor\;region}\times 100$$

## 5. Conclusions

In this work, we have demonstrated that the ERT can be used in the low-conductivity solutions, such as demineralized water. The total variation reconstruction algorithm can be used with two iterations to evaluate a central object in the reactor with an 83-mm diameter covering an area of 1.5% of the reactor area in a static testing environment. The expected accuracy was achieved using the G-Channel segmentations on the reconstructed images. The separability of two objects with a 1.5% area of reactor area was achieved in the demineralized water. Multiple factors have to be accounted for in a quantitative estimation using ERT imaging modality. The discontinuities in the region of interest due to the crystal presence were clearly observed during dynamic testing. It was observed that the total variation algorithm provided good results with G-Channel segmentation, compared to Otsu segmentation, for dynamic evaluations. Variation in the reconstruction parameters for dynamic crystallization studies, compared to static studies, is of interest in future works. There is also a need to develop an interactive human interface software application to observe and analyze the ERT data quickly and to fasten the speed of analysis and calibration. Quantitative analysis can be improved after developing neural network models that consider multiple variables and compare the results with reconstructed images of standardized phantoms. This enables us to quantify the spread of the crystals or locate the dense crystal agglomerations inside the solutions and helps us monitor the growth in the lab-scale chemical reactor.

## Figures and Tables

**Table 1:** Summary of the experiments.

	Experimental Variable	Count
1	Number of solutions with varied conductivities	3
2	Number of phantoms	6
3	Number of reconstruction methods compared	3
4	Number of segmentation methods	4
5	Number of electrodes	16
6	Number of planes	1
7	Minimum accuracy tested	1.5% of the beaker area
8	Location of object	Central and incremental, separability

**Table 2:** Dimensions of the reactor.

	Object	Measured Values mm
1	Batch reactor’s inner diameter	83
2	Electrode tail diameter	5
3	Electrode head diameter	12

**Table 3:** Dimensions of the phantoms.

	Phantom	Measured Values mm	Expected Percentage Area of the Phantom Region (A_P_) %
1	Phantom R1	50 ± 0.1	36.28
2	Phantom R2	40 ± 0.1	23.22
3	Phantom R3	30 ± 0.1	13.06
4	Phantom R4	20 ± 0.1	5.8
5	Phantom R5	10 ± 0.1	1.45
6	Phantom R6	2 × 10 ± 0.1	1.45 and 1.45
5	Diameter of the base of phantoms	50	
7	Distance between centers of phantom R6	40	

## Data Availability

Data available in a publicly accessible repository that does not issue DOIs. This data can be found here: http://bit.ly/39rBIZv.

## Appendix A

Figure A1a–f shows contrast profile plots for the green channel of the image for R1, R2, R3, R4, R5, and R6 at L1.

Figure A2 shows quantitative evaluations of Otsu and G-Channel segmentations after applying E10, E20, and E30 erosion for phantoms in tap water.

**Table A1:** Table showing correlation coefficient of the evaluated percentage area with the phantom diameter and expected area.

							Corr. PD	Corr. A_P_
	Phantom diameter (PD)	10	20	30	40	50		0.9811
	Expected area (A_P_)	1.45	5.81	13.06	23.23	36.29	0.9811	
TV	G-Channel	14.99	17.44	22.10	26.48	38.38	0.9563	0.9904
	Otsu	13.06	17.44	20.69	25.23	35.39	0.9714	0.9920
LBP	G-Channel	16.76	18.53	19.66	21.21	26.16	0.9499	0.9811
	Otsu	10.29	12.22	12.13	13.90	17.76	0.9314	0.9662
GN	G-Channel	7.44	8.28	9.34	10.71	15.38	0.9255	0.9740
	Otsu	4.60	4.70	5.57	6.49	9.23	0.9196	0.9748
	stdevA	4.63	5.69	6.84	8.18	11.59		
	**˄ (%)**	319.14	97.95	52.33	35.20	31.95		

Table A1 shows the quantitative information for tap water along with correlation factors. The standard deviations of the percentage area of phantom regions evaluated by G-Channel and Otsu for all the reconstruction algorithms were calculated using STDEVA function in Microsoft excel. Corr PD shows the correlation coefficient of the evaluated percentage area *A_P_* with the phantom diameters. Corr. EA shows the correlation coefficient of the evaluated percentage area with the expected percentage area. The correlation coefficient was computed using MATLAB function corrcoef(). Factor *˄* was evaluated using the following Equation (A1). Higher *˄* signifies the higher deviations from the expected percentage area *A_P_*.

(A1) $$\wedge =\frac{\mathrm{stdevA}\;}{\mathrm{expected}\;\mathrm{area}\;\mathrm{percentage}\;A_{P}}\times 100$$
